# Evaluation of beliefs, practices and importance among Health Sciences University students regarding hand hygiene

**DOI:** 10.3205/dgkh000595

**Published:** 2025-11-21

**Authors:** Busra Terzioglu, Aslihan Yeniyapı, Elif Aydin, Yesim Tunc, Duygu Percin Renders

**Affiliations:** 1 Kutahya Health Sciences University, Tavsanli Vocational School of Health Services, Kutahya, Turkey; 2 Kutahya Health Sciences University, Oral Health Application and Research Center, Kutahya, Turkey; 3 Agri İbrahim Cecen University, Faculty of Medicine, Department of Medical Microbiology, Agri, Turkey; 4 Kutahya Health Sciences University, Faculty of Medicine, Department of Statistics, Kutahya, Turkey; 5 Kutahya Health Sciences University, Faculty of Medicine, Department of Medical Microbiology, Kutahya, Turkey

**Keywords:** hand hygiene, belief, practice, importance, healthcare

## Abstract

**Aim:**

Healthcare-associated infections are largely preventable and pose a substantial burden on society. The most effective way to control infection is to ensure optimal hand hygiene. This cross-sectional study aimed to evaluate hand hygiene (HH) beliefs, practices, and importance levels among healthcare-professional students.

**Methods:**

Students of Kutahya Health Sciences University, Faculty of Medicine, Faculty of Dentistry, Faculty of Health, and Vocational Schools of Health Services were included in this questionnaire-based study. The questionnaire contained a total of 45 items. The first nine included demographic information; the other items were divided into three subgroups: hand hygiene belief scale (HBS), hand hygiene practice inventory (HHPI), and hand hygiene importance scale (HIS). The questionnaire used a 5-point Likert scale ranging from strongly disagree to strongly agree.

**Results:**

A total of 884 participants (695 female, 189 male) with a mean age of 20.2±2.61(17-49) completed the survey by answering all questions. The mean total scale score of all participants was 145.7±17.89. The mean HBS score was 67.7±10.91, mean HHPI score was 64.7±8.98, and the mean HIS score was 13.4±2.25. It was determined that females and individuals under 20 years old had higher scores than males and participants > 20 years of age, and students in the medical and health sciences departments scored higher than students in vocational schools and the faculty of dentistry.

**Conclusions:**

This study determined that students’ belief, practice, and importance levels regarding the necessity of HH were high and that age, gender, and university department affected HH compliance.

## Zusammenfassung

**Zielsetzung:**

Health care assoziierte Infektionen sind zu einem großen Teil vermeidbar und stellen eine erhebliche Belastung für die Gesellschaft dar. Die wirksamste Methode zur Infektionskontrolle ist die Gewährleistung optimaler Händehygiene. Ziel der Querschnittsstudie war es, Überzeugungen, Praktiken und Wichtigkeitsgrade der Händehygiene von Kandidaten für den Beruf im Gesundheitswesen zu bewerten.

**Methoden:**

An der Studie nahmen Studierende der Kutahya Health Sciences University, der Fakultät für Medizin, der Fakultät für Zahnmedizin, der Fakultät für Gesundheit und der Berufsschulen für Gesundheitsdienste teil. Der in der Studie verwendete Fragebogen enthielt 45 Fragen. Dabei enthielten die ersten neun Fragen demografische Informationen. Die anderen Fragen beinhalteten drei Untergruppen: Händehygiene-Überzeugungsskala (HÜS), Händehygiene-Praxisinventar (HPI) und Händehygiene-Wichtigkeitsskala (HWS). Der Fragebogen ist eine 5-Punkte-Likert-Skala, wobei jede Aussage von „Stimme überhaupt nicht zu“ bis „Stimme völlig zu“ reicht.

**Ergebnisse:**

884 Teilnehmer (695 Frauen, 189 Männer) mit einem Durchschnittsalter von 20,2±2,61 (17–49) Jahren haben die Umfrage abgeschlossen und alle Fragen beantwortet. Der durchschnittliche Gesamtskalenwert aller Teilnehmer betrug 145,7±17,89. Der durchschnittliche HÜS-Wert betrug 67,7±10,91, der durchschnittliche HPI-Wert 64,7±8,98 und der durchschnittliche HWS-Wert 13,4±2,25. Es wurde festgestellt, dass Frauen und Personen unter 20 Jahren höhere Werte erzielten, während Studenten der medizinischen und gesundheitswissenschaftlichen Fakultäten bessere Werte erzielten als Studenten der Berufsschulen und der zahnmedizinischen Fakultät.

**Schlussfolgerungen:**

Die Studie ergab, dass die Überzeugung, Praxis und Wichtigkeit der Studenten in Bezug auf die Notwendigkeit von Händehygiene hoch waren und dass Alter, Geschlecht und Universitätsabteilung die Einhaltung der Händehygiene beeinflussten.

## Introduction

To a great extent, healthcare-associated infections (HAIs) are preventable, but their morbidity and mortality pose a substantial burden on society [1], [2]. It has been estimated that hundreds of thousands of individuals are impacted by avoidable HAIs annually [3]. Various factors contribute to the occurrence of HAIs, stemming from deficiencies in health-related policies, infrastructure, organization, and knowledge [4]. Additionally, substandard practices and behaviors among healthcare professionals further exacerbate the risk of HAIs. The transmission of HAIs is primarily attributed to the contaminated hands of healthcare workers [5]. As a result, upholding high standards of hand hygiene (HH) has always been a paramount concern in the healthcare sector [6]. Despite the seemingly straightforward nature of HH, achieving and maintaining compliance with HH protocols in healthcare settings has consistently presented significant challenges on a global scale [7]. According to the literature, the average HH compliance rate is approximately 40% in high-income countries and less than 20% in low-income countries [8]. A study revealed that a mere 5.55% of medical and nursing students adhered to proper HH practices, indicating a prevalent lack of compliance among students [9].

Studies have identified various factors associated with low HH compliance within healthcare settings. It has been reported that inadequate staffing and overcrowding are linked to reduced HH adherence among healthcare workers, leading to an increase in HAIs caused by methicillin-resistant *Staphylococcus aureus* [10]. It has been stated that the place of duty and the workload of healthcare personnel are related to HH compliance [6]. In a study that monitored the HH practices of medical students upon entering intensive care units, it was observed that male students often neglected HH due to perceived time constraints, lack of role models for proper HH, and misinformation about the requirements [11]. Conversely, female students cited concerns about skin dryness or cracking and forgetfulness as reasons for non-compliance [11]. In various studies investigating the HH knowledge and attitudes of nursing and medical students, it was found that nursing students consistently exhibited higher compliance with HH practices [9], [12], [13], [14], [15]. This disparity has been attributed to differences in the timing of HH training between the two cohorts and the frequency of direct patient contact. Recent reports indicate that following HH protocols is linked to glove usage. Unnecessary glove use has negative consequences, while appropriate glove use has positive effects [12].

The primary focus of the literature on student HH compliance predominantly centers around medical and nursing students. Given the widespread inadequacy of HH compliance globally, it is essential to explore the knowledge and attitudes of students across all healthcare disciplines regarding HH practices.

The main purpose of this study was to evaluate the beliefs, practices, and importance of HH among healthcare-professional students. Additionally, the study seeks to assess the potential impact of various associated factors, including age, gender, university department, and HH training.

## Methods

### Study design and setting

In this descriptive, cross-sectional study, a survey form was used to determine the beliefs, practice, and importance of HH in students training to be health technicians and doctors, dentists, nurses, midwives, physiotherapists, and dieticians. The survey form was sent to participants online via a link or e-mail. The data collection process continued between January 2024 and April 2024, and the collected data were recorded in a database. Ethical approval was obtained from Kutahya Health Sciences University Non-Interventional Clinical Research Ethics Committee on 15.01.2024 (decision number 2024/01-34).

### Participants

Students of the Kutahya Health Sciences University, Faculty of Medicine, Faculty of Dentistry, Faculty of Health Sciences (midwifery, nursing, physiotherapy, nutrition and dietetics), and Vocational Schools of Health Services (oral and dental health technician, emergency and first aid, medical laboratory technician, physiotherapy technician, dental prosthesis technician, anesthesia technician, patient care, elderly care, medical imaging technician, disinfection/sterilization technician, occupational therapy technician) were included in the study. Students outside the specified universities and departments were excluded. 

Participants were categorized into four groups according to their department: Vocational Schools of Health Services (Group 1), Faculty of Health Sciences (Group 2), Faculty of Dentistry (Group 3), and Faculty of Medicine (Group 4).

### Data sources

The questionnaire contained a total of 45 items. The first 9 included demographic information: age, gender, faculty, program, class, type of highschool diploma, HH education, source of HH education, and the institution in which they practice. The other questions were divided into three subgroups: hand hygiene belief scale (HBS), hand hygiene practice inventory (HHPI), and hand hygiene importance scale (HIS). The HBS comprises 19 questions and assesses the belief in the necessity of HH application. The HHPI consists of 14 questions and evaluates HH application in hospital settings. The HIS contains 3 questions and gauges opinions on the importance of HH. The questionnaire was answered on a 5-point Likert scale with each statement ranging from strongly disagree (1 point) to strongly agree (5 points). The scale ranges from 36 to 180, with no specified cut-off point. The HBS scores range from 19 to 95, the HHPI scores range from 14 to 70, and the HIS scores range from 3 to 15. As the score on the scale increases, it indicates that the participants’ knowledge, beliefs, and practices related to HH increase. The Cronbach alpha values for the scale were 0.80 for HBS, 0.74 for HHPI, and 0.77 for HIS [16]. The Turkish questionnaire’s validity and reliability were also proven, and was presented to the students in their mother tongue [17]. Participants chose one or more options depending on the design of the questions. It took approximately 20 minutes to complete the survey by answering the questions.

### Outcomes

Primary outcomes were investigation of HH knowledge, beliefs, practices, and importance levels of students at a health sciences university. Secondary outcomes were examining the effect of the university department and gender, age, and HH education status on HBS, HHPI, and HIS scores and total scale score. 

### Sample size

Assuming an error level of 5%, a confidence level of 99%, and an estimated response rate of 50%, the number of people to be invited was 1172 and the required sample size 586. The study invited all students (n=5390) from the faculty of medicine, faculty of health sciences, and vocational schools of health services. A total of 884 students participated in the study and completed the questionnaire, resulting in a response rate of 16.4%.

### Statistical analysis

Descriptive statistics of the data, including number, percentage, mean, standard deviation, minimum, and maximum were provided. To begin the statistical analysis, the assumption of normality was checked using Skewness & Kurtosis, the Kolmogorov-Smirnov test, and a histogram. Because the normality assumption was not met, the Mann-Whitney U-test was used. The Kruskal-Wallis test was used for variables with three or more independent groups that did not exhibit a normal distribution. The significance level was set at 0.05. Analyses were carried out in the IBM SPSS 25 program.

## Results

### Demographic data

A total of 884 participants (695 female, 189 male) with a mean age of 20.2±2.61 (17–49) years completed the survey by answering all questions. 42.5% of the participants were studying at vocational schools of health services, 35.9% at a University health-sciences department, 16.5% at a University faculty of dentistry, and 5.1% at a University faculty of medicine. 64.9% of the participants stated that they had received HH training, while 35.1% stated that they had not (Table 1 (Tab. 1)). 

### Evaluation of survey questions

The mean total scale score of all participants was 145.7±17.89. The mean HBS score was 67.7±10.91, the mean HHPI score was 64.7±8.98, and the mean HIS score was 13.4±2.25 (Table 2 (Tab. 2)).

The statements with the highest “strongly agree” rates were “Cleansing hands after going to the toilet can reduce transmission of infectious disease” (HBS-19) (71.2%), “I follow the example of senior healthcare workers when deciding whether or not to perform hand hygiene” (HBS-6) (62.2%) and “Performing hand hygiene after wound care can protect from infections” (HBS-18) (61.5%). On the other hand, the statements with the highest “strongly disagree” rates were “When busy it is more important to complete my tasks than to perform hand hygiene” (HBS-2) (41.0%), “I can’t always perform hand hygiene in recommended situations because my patient’s needs come first” (HBS-5) (41.0%). Moreover, 20.9% of the participants strongly agreed with the “HBS-1” question, 52.1% with the “HBS-3” question, and 52.1% with the “HBS-4” question (see Table 3 (Tab. 3)). Further, 21.4% strongly agreed with the “HBS-7” question, 17.9% with the “HBS-8” question, 42.2% with the “HBS-9” question, 59.8% with the “HBS-10” question, and 49.7% with the “HBS-11” question (Table 3 (Tab. 3)).

In the question “I cleanse my hands…”, the statements with the highest “strongly agree” rates were “After going to the toilet” (HHPI-1) (83.6%) and “After contact with blood or body fluids” (HHPI-6) (81.4%), respectively. The statements with the lowest “strongly agree” rates were “Before entering an isolation room” (HHPI-8) (67.9%) and “Before patient contact” (HPPI-13) (67.3%). The statements with the highest “strongly disagree” rates were “After caring for a wound” (HHPI-3) (1.7%) and “After removing gloves” (HPPI-14) (1.2%) (Table 4 (Tab. 4)).

While 54.8% of the participants responded “strongly agree” to HIS-1, 31.2% responded “agree”. 63.3% responded “strongly agree” to HIS-2, while 29.3% responded “agree”. Furthermore, 61.9% responded “strongly agree” to HIS-3, while 29.9% responded “agree” (Table 3 (Tab. 3)).

### Influence of demographic data on HBS, HHPI, HIS and total scale scores

The effect of gender, age, and HH training on HBS, HHPI, HIS, and total scale scores is presented in Table 4 (Tab. 4). It was observed that HHPI, HIS, and total scale scores were significantly higher in women than in men (p=0.000, p=0.002, p=0.005, resp.). However, there was no statistically significant difference between the two genders in the HBS score (p=0.997). It was observed that HBS, HHPI, and total scale scores were significantly higher in the age groups <20 years compared to the age groups ≥20 years (p=0.037, p=0.026, p=0.010). However, there was no statistically significant difference between the two age groups in the HIS score (p=0.450). When the effect of HH training on HBS, HHPI, HIS, and total scale scores was examined, only HIS scores were found to be significantly higher in the group that received HH training (p=0.006). No statistically significant difference was found in HBS, HHPI, and total scale scores between participants who received and did not receive HH training (p=0.450).

### Influence of the university department on HBS, HHPI, HIS, and total scale scores

In terms of the influence of university department on HBS, HHPI, HIS, and total scale scores, statistically significant differences were found among the four groups (Table 5 (Tab. 5)). When comparing HBS scores, group 2 was found to be higher than groups 1 and 3 (p=0.00, p=0.00). There was no significant difference between the other groups (p>0.05). In terms of HHPI scores, group 2 scored higher than group 3 (p=0.00), and no significant difference was found between the other groups (p>0.05). When comparing HIS scores, group 2 scores were found to be higher than those of groups 1 and 3 (p=0.00, p=0.00), with no significant difference found between the other groups (p> 0.05). Furthermore, when comparing total scale scores, group 2 had higher scores than groups 1 and 3 (p=0.00, p=0.00), and group 4 scores were found to be higher than those of group 3 (p=0.007). No significant difference was found between the other groups (p>0.05). When comparing the mean ages of these four groups, it was observed that groups 1 and 2 had lower mean ages compared to groups 3 and 4 (p=0.00, p=0.00, p=0.00, and p=0.00) (Table 5 (Tab. 5)).

## Discussion

Ensuring HH is the most effective way to infection control. This study evaluated the HH beliefs, practices, and importance levels of students at a health sciences university and examined the effect of demographic data. Our results showed that demographic data such as age, gender, university department, and HH training impact HH beliefs, practices, and importance. 

In this study, the HHPI score was quite high (mean 64.7±8.98), consistent with other studies conducted in Turkey [18], [19], [20]. Although the average HBS score (mean 67.7±10.91) was lower than in the study by Birgili et al. [21], our study had similar HIS (mean 13.4±2.25) and high HHPI and total scale scores (145.7±17.89). Unlike this study, HBS in some similar studies in the literature consisted of 22 questions, with a possible score range of 22-110. In those studies, the average HBS score of nursing students was reported as 86.4 ± 8.56 and 89.8±7.98 [18], [20]. 

This study revealed that gender has an impact on HH, with women achieving higher scores on HHPI, HIS, and the total scale. Multiple studies have indicated that female participants tend to have higher scores, and research on HH compliance has also shown that female healthcare workers demonstrate higher levels of compliance [18], [22], [23], [24]. In contrast, one study reported higher HHBS and HHPI scores in male participants compared to females [20], and another study indicated no effect of gender [21]. These findings suggest the need for tailored interventions to address gender-specific barriers to HH adherence within healthcare settings. The difference between genders in this study may have been because most participants were women and men were not adequately represented.

In this study, students under 20 had higher HBS, HHPI, and total scale scores. In this study, where the population consisted of students, although the students were close in age, this significant difference shows that the younger population takes HH more seriously. In contrast, a study on young health professional candidates from Generation Z reported that age had no significant effect [22]. In addition, several studies have reported a positive correlation between increasing age and HH scale scores [23], [25].

Unlike other studies in the literature, this study, which included all health-professional students at a health sciences university, revealed that vocational schools and dentistry students had lower HBS, HHPI, HIS, and total scale scores compared to medical and health-sciences faculty students. It has been shown that the importance given to HH in health-sciences departments and medical faculty curricula is not given in vocational schools and dentistry faculties. The brevity of training (two years) in vocational schools and the absence of invasive treatment attempts with patients in some departments may have affected the low scores. However, considering the duration of dentistry education (five years) and the internship periods (two years) where one-on-one contact with the patient is provided, it should be possible to make improvements in the dental curriculum regarding HH. In addition, inadequate knowledge, attitudes, and practices regarding HH have been reported in studies focusing on dental students or dentists [26], [27], [28]. In some studies comparing medical and nursing students, nursing students were reported to have higher scores, while in other studies, medical students were reported to have higher scores [9], [14], [15], [29], [30], [31]. In this study, no difference was found between health-sciences students and medical students, which is consistent with studies in which medical and nursing students had similar scores. Similar to this study, Baier et al. [22] reported that dental students had lower HH knowledge than medical technical assistants and trainee nurses. In the study by Thakker et al. [30], medical students had better HH knowledge than dental and nursing students.

The analysis showed that HH training could impact students’ beliefs about and importance attributed to HH, as well as their practice scores. It was found that the training was effective in achieving higher scores in all subgroups and total scale scores, but the difference was only significant in the HIS score. The fact that HH training did not cause a significant difference in the effective total scale score may have been due to the information campaigns conducted worldwide through mass media during the COVID-19 pandemic and the increase in public awareness.

In this study, the participants’ highest level of agreement in providing HH was determined as “After going to the toilet” and “After contact with blood or body fluids”, and this data is consistent with previous studies [18], [24]. In addition, the lowest level of agreement was determined as “Before patient contact” and “Before entering an isolation room”. This is similar to the study of Nicholson et al. [32], which reported that healthcare professionals are more likely to wash their hands after patient contact rather than before. Unfortunately, the fact that students did not realize the necessary importance of HH before patient contact suggests that the healthcare professional’s responsibility to protect the patient is not fully established. In addition, in this study, the statement with the highest level of agreement, consistent with the literature, is “Cleansing hands after going to the toilet can reduce transmission of infectious disease” [18], [24].

### Limitations

The main limitation of this study was the risk of response bias and overestimation due to its online survey-based nature. It was also conducted at a single health-sciences university in Turkey and these findings may not be generalizable to different populations. In addition, although the required sample size was reached, attempts were made to reach all students and the questionnaire was sent twice at 2-week intervals as a reminder, the participation rate remained low. Another limitation of the study is that the number of female and male participants and the number of participants between departments could not be equal because the survey was volunteer-based. In addition, studies that monitor HH practices and compare them with the reported responses may be valuable in revealing students’ HH beliefs, practices, and importance levels.

## Conclusion

This study revealed that the beliefs about the necessity of HH, the practice thereof, and the importance attributed to HH among health-professional students received high scores. However, enhancing the role of HH education in the curriculum, as well as emphasizing its necessity, particularly in departments with lower average hand-hygiene scores, may be beneficial. It was also determined that age, gender, and university department affect HH compliance. Therefore, personalizing HH training according to demographic characteristics may be valuable.

## Notes

### Authors’ ORCIDs

- Busra Terzioglu: 0000-0002-8534-6718
- Aslihan Yeniyapi: 0000-0002-0105-9229
- Elif Aydin: 0000-0003-0877-453X
- Yesim Tunc: 0000-0002-1078-8730
- Duygu Percin Renders: 0000-0002-4436-5226

### Ethical approval 

Ethics committee approval was obtained from Kutahya Health Sciences University Non-Interventional Clinical Research Ethics Committee on 15.01.2024 (decision number 2024/01-34)

### Funding

None. 

### Competing interests

The authors declare that they have no competing interests.

## Figures and Tables

**Table 1:**
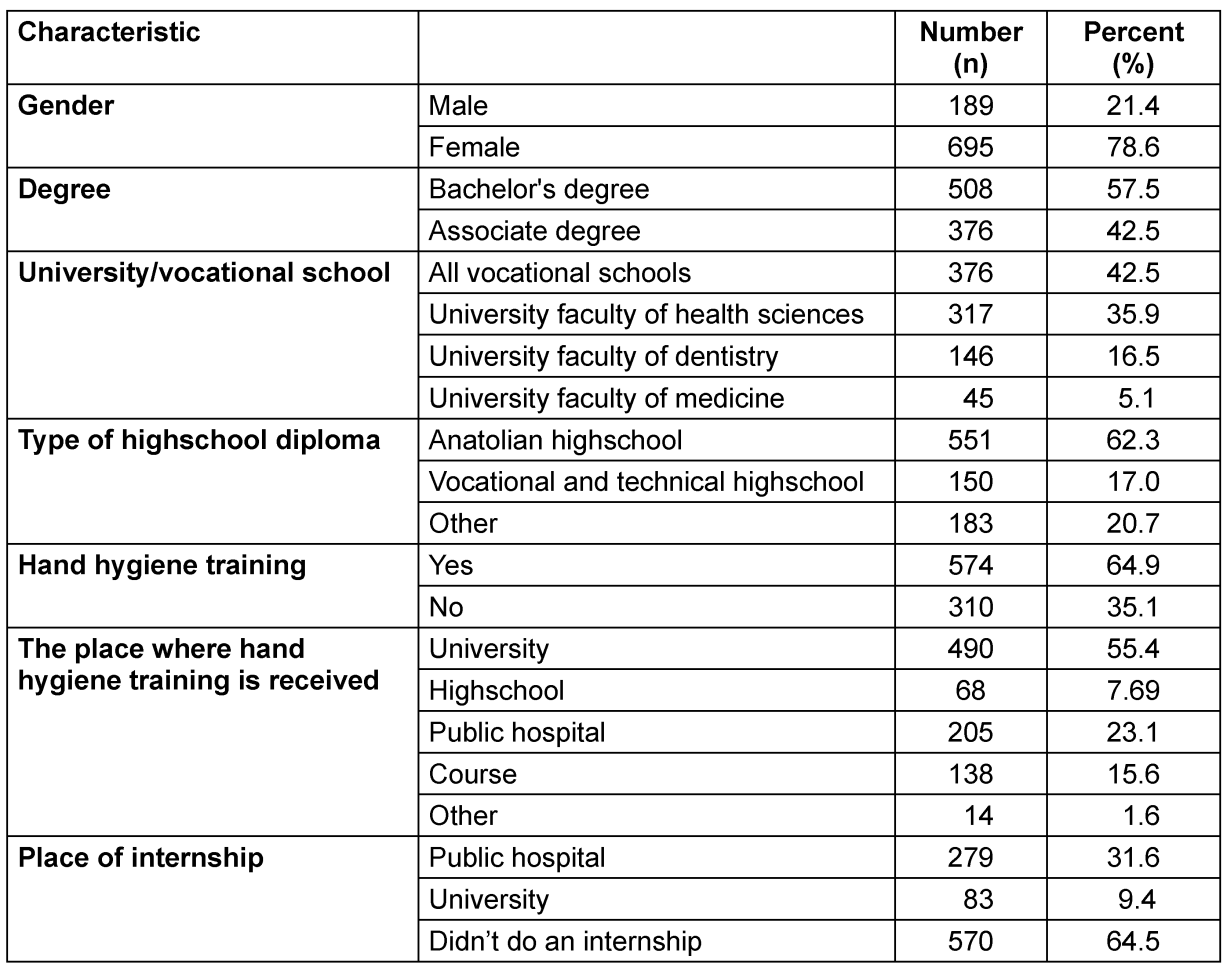
Demographic characteristics of the participants

**Table 2:**
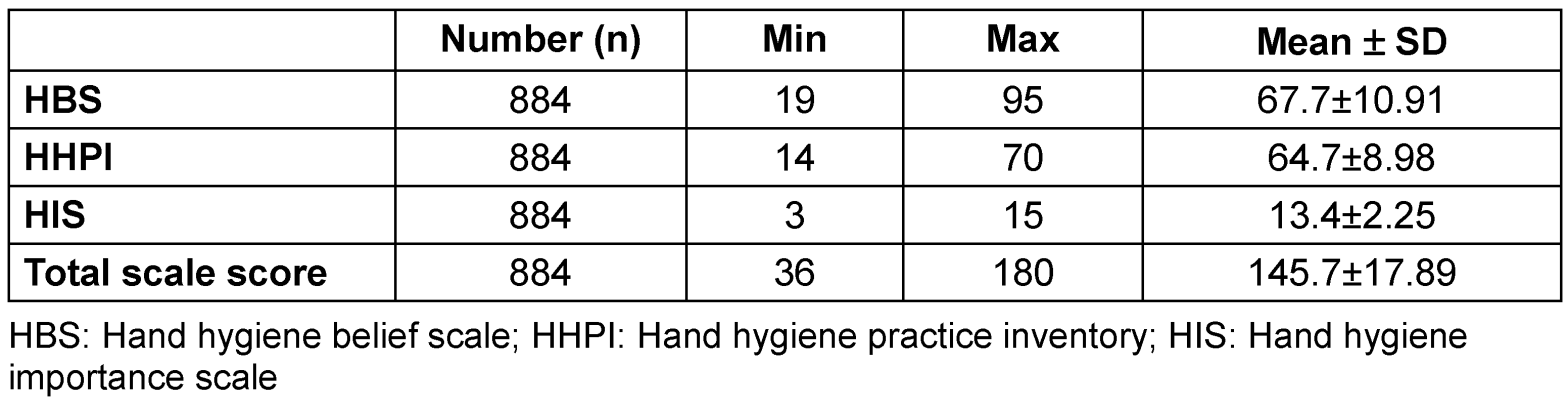
Distribution of the different hand-hygiene scores

**Table 3:**
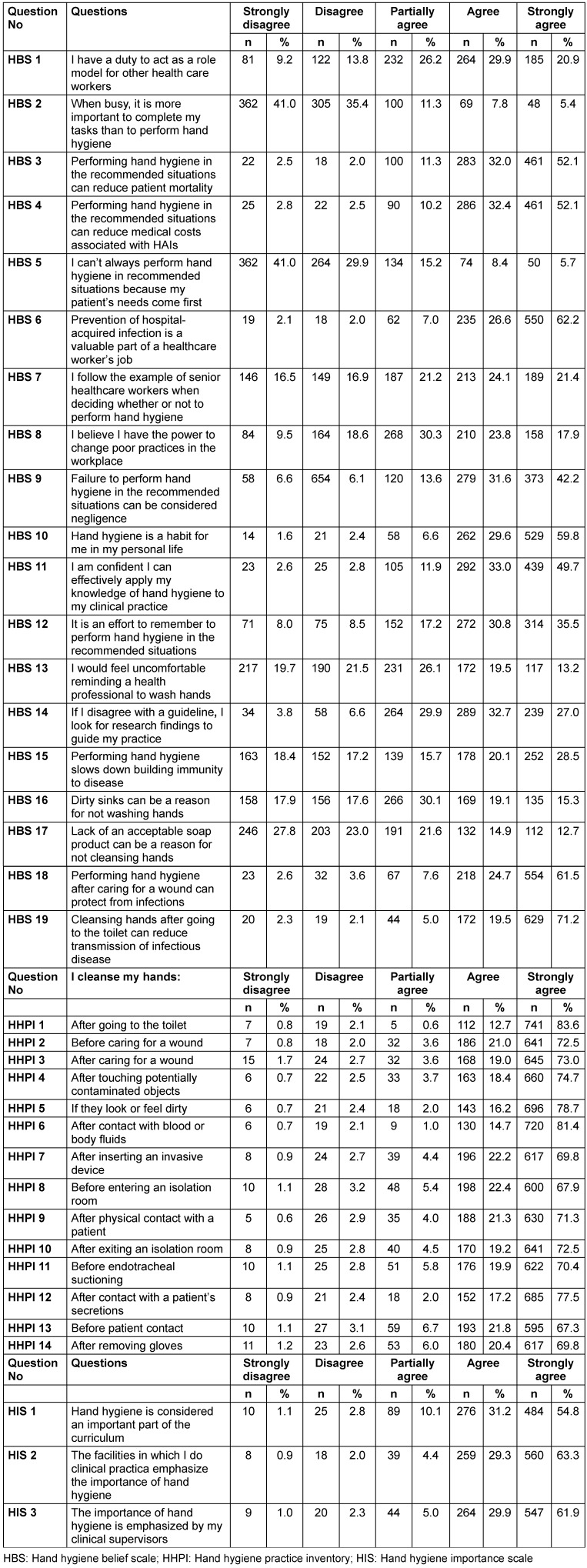
Distribution of participants according to their answers to hand hygiene belief, hand hygiene practice inventory and hand hygiene importance

**Table 4:**
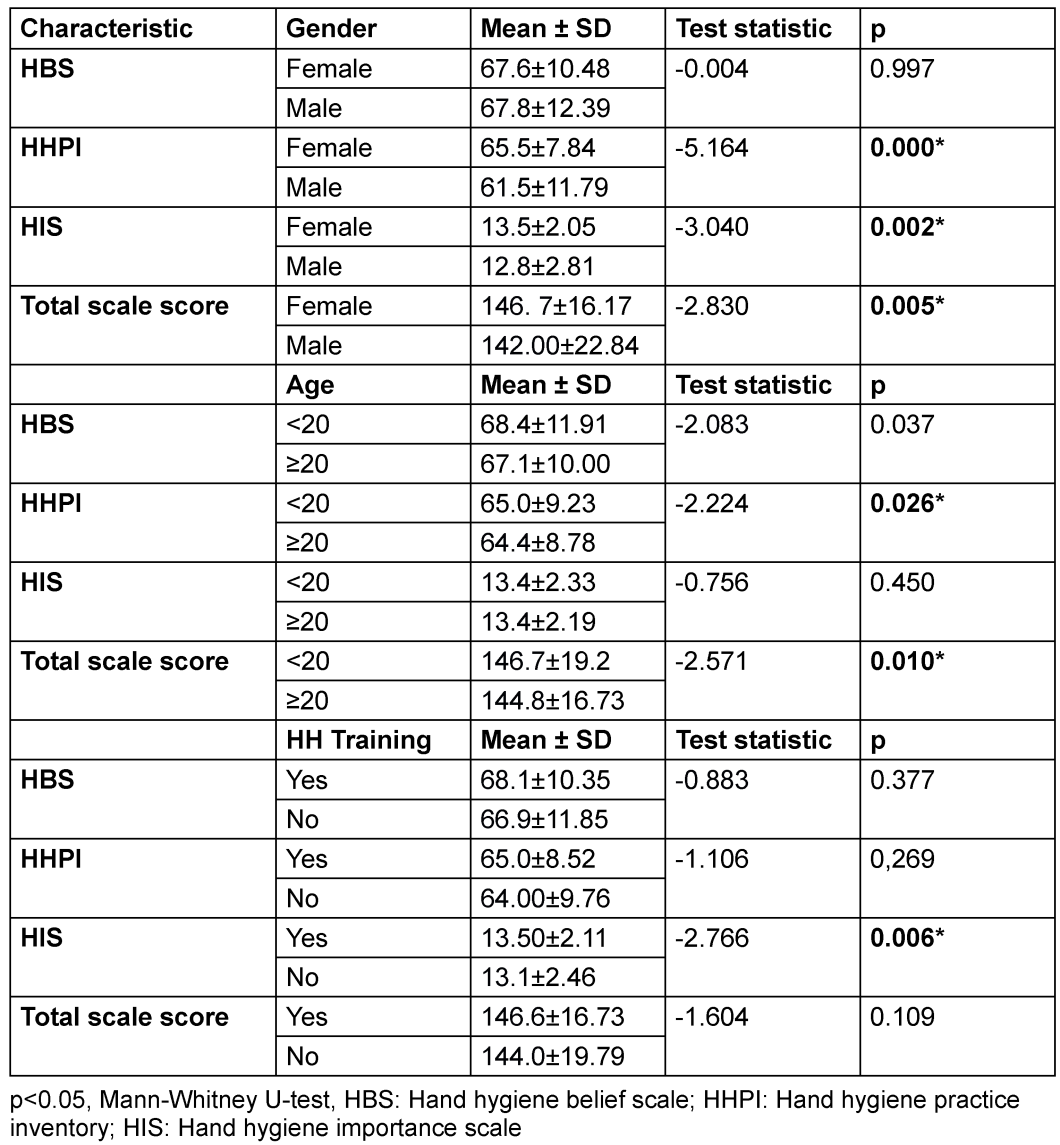
Influence of gender, age, and HH training on hand hygiene belief scale, hand hygiene practice inventory, hand hygiene importance scale and total scale scores

**Table 5:**
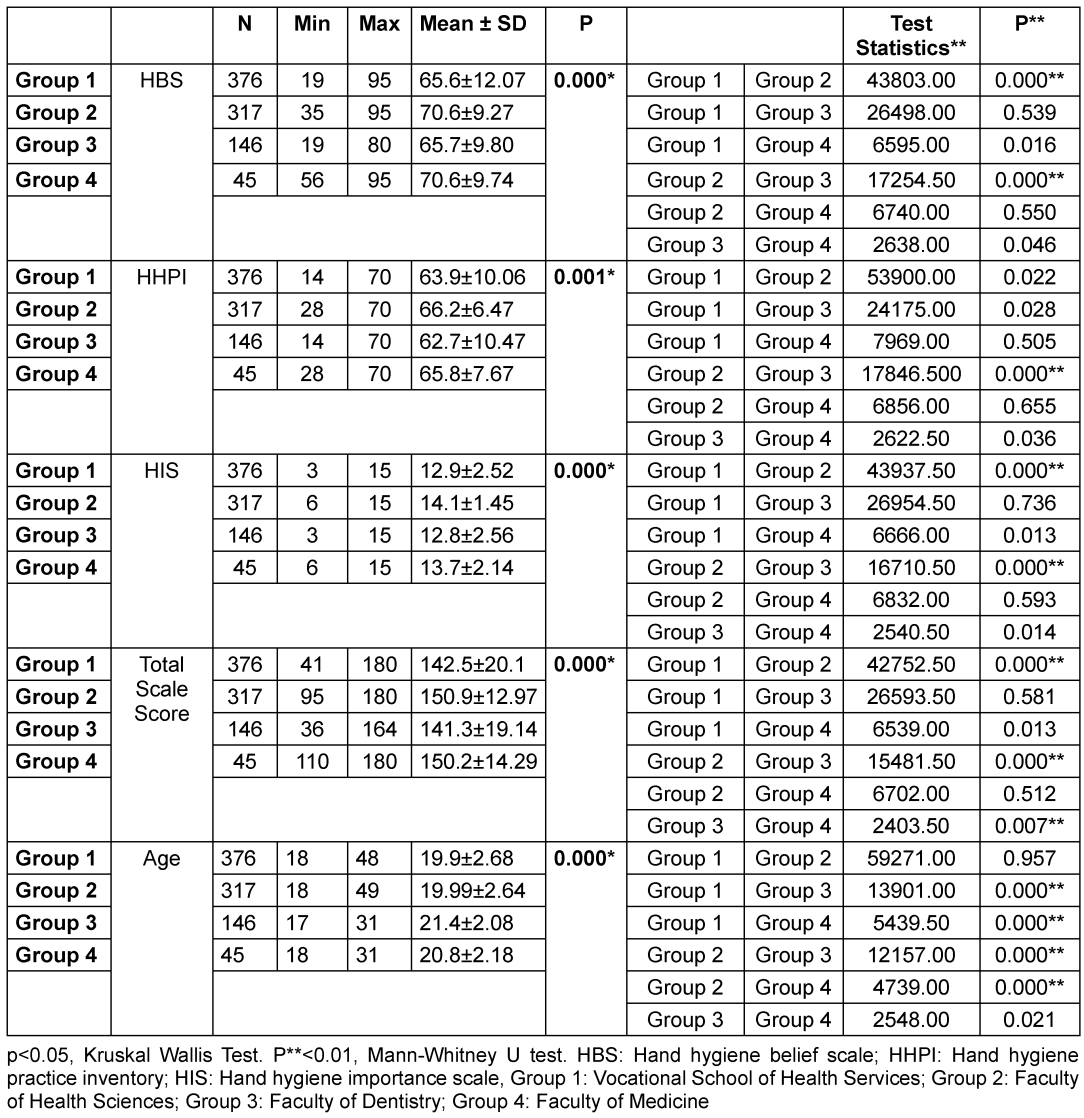
Influence of university department on hand hygiene belief scale, hand hygiene practice inventory, hand hygiene importance scale and total scale scores

## References

[1] WHO (2011). Report on the burden of endemic health care-associated infection worldwide.

[2] Revelas A (2012). Healthcare - associated infections: A public health problem. Niger Med J.

[3] Maki G, Zervos M (2021). Health Care-Acquired Infections in Low- and Middle-Income Countries and the Role of Infection Prevention and Control. Infect Dis Clin North Am.

[4] Vandijck D, Cleemput I, Hellings J, Vogelaers D (2013). Infection prevention and control strategies in the era of limited resources and quality improvement: a perspective paper. Aust Crit Care.

[5] Toney-Butler TJ, Gasner A, Carver N (2023). Hand Hygiene.

[6] Pittet D, Allegranzi B, Sax H, Dharan S, Pessoa-Silva CL, Donaldson L, Boyce JM, WHO Global Patient Safety Challenge, World Alliance for Patient Safety (2006). Evidence-based model for hand transmission during patient care and the role of improved practices. Lancet Infect Dis.

[7] Lotfinejad N, Peters A, Tartari E, Fankhauser-Rodriguez C, Pires D, Pittet D (2021). Hand hygiene in health care: 20 years of ongoing advances and perspectives. Lancet Infect Dis.

[8] Allegranzi B, Bagheri Nejad S, Combescure C, Graafmans W, Attar H, Donaldson L, Pittet D (2011). Burden of endemic health-care-associated infection in developing countries: systematic review and meta-analysis. Lancet.

[9] Ariyaratne M, Gunasekara T, Weerasekara M, Kottahachchi J, Kudavidanage B, Fernando S (2013). Knowledge, attitudes and practices of hand hygiene among final year medical and nursing students at the University of Sri Jayewardenepura. 2015. Sri Lankan Journal of Infectious Diseases.

[10] Clements A, Halton K, Graves N, Pettitt A, Morton A, Looke D, Whitby M (2008). Overcrowding and understaffing in modern health-care systems: key determinants in meticillin-resistant Staphylococcus aureus transmission. Lancet Infect Dis.

[11] Birnbach DJ, Rosen LF, Fitzpatrick M, Arheart KL, Everett-Thomas R (2019). Current hand hygiene education is suboptimal. Clin Teach.

[12] Baccolini V, D'Egidio V, de Soccio P, Migliara G, Massimi A, Alessandri F, Tellan G, Marzuillo C, De Vito C, Ranieri MV, Villari P (2019). Effectiveness over time of a multimodal intervention to improve compliance with standard hygiene precautions in an intensive care unit of a large teaching hospital. Antimicrob Resist Infect Control.

[13] Le CD, Lehman EB, Nguyen TH, Craig TJ (2019). Hand Hygiene Compliance Study at a Large Central Hospital in Vietnam. Int J Environ Res Public Health.

[14] van de Mortel TF, Kermode S, Progano T, Sansoni J (2012). A comparison of the hand hygiene knowledge, beliefs and practices of Italian nursing and medical students. J Adv Nurs.

[15] Kingston LM, O'Connell NH, Dunne CP (2018). A comparative study of hand hygiene and alcohol-based hand rub use among Irish nursing and medical students. Nurse Educ Today.

[16] van de Mortel T (2009). Development of a questionnaire to assess health care students' hand hygiene knowledge, beliefs and practices. Austral J Advanc Nursing.

[17] Birgili F, Baybuga M, Ozkoc H, Kuru O, van de Mortel T, Tümer A (2019). Validation of a Turkish translation of The Hand Hygiene Questionnaire. East Mediterr Health J.

[18] Ceylan B, Gunes U, Baran L, Ozturk H, Sahbudak G (2020). Examining the hand hygiene beliefs and practices of nursing students and the effectiveness of their handwashing behaviour. J Clin Nurs.

[19] Alcan AO, Dolgun E (2019). Student nurses' hand hygiene beliefs and practices. Turkish J Family Med Primary Care.

[20] Karadag M, Iseri OP, Yildirim N, Etikan I (2016). Knowledge, beliefs and practices of nurses and nursing students for hand hygiene. Jundishapur J Health Sci.

[21] Birgili F, Yazkan G, Bulut Ugurlu N (2023). Pandemi Döneminde Hemşirelerin El Hijyeni Bilgi, İnanç ve Uygulamalarının Değerlendirilmesi: Tanımlayıcı Araştırma. Turkiye Klinikleri J Nurs Sci.

[22] Baier C, Albrecht UV, Ebadi E, Vonberg RP, Schilke R (2020). Knowledge about hand hygiene in the Generation Z: A questionnaire-based survey among dental students, trainee nurses and medical technical assistants in training. Am J Infect Control.

[23] Al-Qahtani AM (2023). Clean hands, safe care: how knowledge, attitude, and practice impact hand hygiene among nurses in Najran, Saudi Arabia. Front Public Health.

[24] Sahiner P (2024). Is there a relationship between nurses' hand hygiene beliefs, practices and ethical sensitivity? Appl Nurs Res.

[25] Goodarzi Z, Haghani S, Rezazade E, Abdolalizade M, Khachian A (2020). Investigating the Knowledge, Attitude and Perception of Hand Hygiene of Nursing Employees Working in Intensive Care Units of Iran University of Medical Sciences, 2018-2019. Maedica (Bucur).

[26] Myers R, Larson E, Cheng B, Schwartz A, Da Silva K, Kunzel C (2008). Hand hygiene among general practice dentists: a survey of knowledge, attitudes and practices. J Am Dent Assoc.

[27] Abreu MH, Lopes-Terra MC, Braz LF, Rímulo AL, Paiva SM, Pordeus IA (2009). Attitudes and behavior of dental students concerning infection control rules: a study with a10-year interval. Braz Dent J.

[28] Thivichon-Prince B, Barsotti O, Girard R, Morrier JJ (2014). Hand hygiene practices in a dental teaching center: Measures and improve. Eur J Dent.

[29] Bouget Mohammedi S, Landelle C (2023). Review of literature: Knowledge and practice of standard precautions by nursing student and teaching techniques used in training. Am J Infect Control.

[30] Thakker VS, Jadhav PR (2015). Knowledge of hand hygiene in undergraduate medical, dental, and nursing students: A cross-sectional survey. J Family Med Prim Care.

[31] Labrague LJ, McEnroe-Petitte DM, van de Mortel T, Nasirudeen AMA (2018). A systematic review on hand hygiene knowledge and compliance in student nurses. Int Nurs Rev.

[32] Nicholson AM, Tennant IA, Martin AC, Ehikhametalor K, Reynolds G, Thoms-Rodriguez CA, Nagassar R, Hoilett TK, Allen R, Redwood T, Crandon I (2016). Hand hygiene compliance by health care workers at a teaching hospital, Kingston, Jamaica. J Infect Dev Ctries.

